# cgMLST characterisation of invasive *Neisseria meningitidis* serogroup C and W strains associated with increasing disease incidence in the Republic of Ireland

**DOI:** 10.1371/journal.pone.0216771

**Published:** 2019-05-29

**Authors:** Robert M. Mulhall, Desiree E. Bennett, Holly B. Bratcher, Keith A. Jolley, James E. Bray, Piaras P. O’Lorcain, Suzanne M. Cotter, Martin C. J. Maiden, Robert J. Cunney

**Affiliations:** 1 Irish Meningitis and Sepsis Reference Laboratory, Temple Street Children’s University Hospital, Dublin, Ireland; 2 Department of Zoology, University of Oxford, Oxford, England, United Kingdom; 3 Health Protection and Surveillance Centre, Dublin, Ireland; 4 Department of Microbiology, Royal College of Surgeons in Ireland, Dublin, Ireland; Universidad Nacional de la Plata, ARGENTINA

## Abstract

**Introduction and aims:**

Since 2013 MenC and MenW disease incidence and associated mortality rates have increased in the Republic of Ireland. From 2002/2003 to 2012/2013, the average annual MenC incidence was 0.08/100,000, which increased to 0.34/100,000 during 2013/2014 to 2017/18, peaking in 2016/17 (0.72/100,000) with an associated case fatality rate (CFR) of 14.7%. MenW disease incidence has increased each year from 0.02/100,000 in 2013/2014, to 0.29/100,000 in 2017/18, with an associated CFR of 28.6%. We aimed to characterise and relate recent MenC isolates to the previously prevalent MenC:cc11 ET-15 clones, and also characterise and relate recent MenW isolates to the novel ‘Hajj’ clones.

**Methods:**

Using WGS we characterised invasive (n = 74, 1997/98 to 2016/17) and carried (n = 16, 2016/17) MenC isolates, and invasive (n = 18, 2010/11 to 2016/17) and carried (n = 15, 2016/17) MenW isolates. Genomes were assembled using VelvethOptimiser and stored on the PubMLST *Neisseria* Bacterial Isolate Genome Sequence Database. Isolates were compared using the cgMLST approach.

**Results:**

Most MenC and MenW isolates identified were cc11. A single MenC:cc11 sub-lineage contained the majority (68%, n = 19/28) of recent MenC:cc11 disease isolates and all carried MenC:cc11 isolates, which were interspersed and distinct from the historically significant ET-15 clones. MenW:cc11 study isolates clustered among international examples of both the original UK 2009 MenW:cc11, and novel 2013 MenW:cc11clones.

**Conclusions:**

We have shown that the majority of recent MenC disease incidence was caused by strain types distinct from the MenC:cc11 ET-15 clone of the late 1990s, which still circulate but have caused only sporadic disease in recent years. We have identified that the same aggressive MenW clone now established in several other European countries, is endemic in the RoI and responsible for the recent MenW incidence increases. This data informed the National immunisation Advisory Committee, who are currently deliberating a vaccine policy change to protect teenagers.

## Introduction

The Gram-negative diplococcus *Neisseria meningitidis* is normally a harmless coloniser of the upper respiratory tract. On rare occasions the meningococcus can cross the nasopharyngeal epithelium and cause invasive meningococcal disease (IMD), the onset and progression of which is often rapid. Associated mortality ranges between 10%-40%, and many survivors experience long term serious sequelae, including hearing impairment and limb loss [1].

The capsular polysaccharide defines the serogroup, and is considered the most important virulence determinant [2]. In Europe, most disease incidence is caused by meningococcal strains expressing serogroups B, C, W, or Y, to which vaccines are available [3].

Multi locus enzyme electrophoresis (MLEE), and later multi locus sequence typing (MLST), revealed meningococcal populations to exist as groups of genetically related clones termed electrophoretic types and clonal complexes (cc) respectively [4,5]. Whole genome sequencing (WGS) has been increasingly used over the past 10 years to resolve meningococcal populations into lineages with epidemiological consequence using a subset of meningococcal core genes [6]. Specific genetic lineages of the meningococcus can be associated with either asymptomatic carriage, which is highest in young adults, or with invasive disease, where the highest rates occur in those under 5 years and young adults [7]. Just a few lineages, the hyper-invasive lineages, are consistently isolated from invasive disease cases, and are responsible for the vast majority of IMD globally [4].

Of particular importance is the hyper-invasive cc11 meningococcal lineage (lineage 11) which are associated with high levels of morbidity and mortality, and can express serogroups C, W, B or Y [8–10]. The epidemiology of cc11 meningococci ranges from sporadic disease cases and clusters, to regional outbreaks (which can be protracted) and international epidemics [11–13].

The first glycoconjugate vaccines were used to combat an international epidemic associated with MenC:cc11 meningococci, which spread through Europe during the mid to late 1990s [13]. Since the early 2000’s several small clusters of MenC:cc11 meningococci have been reported in men who have sex with men (MSM), and recently a protracted regional MenC:cc11 outbreak was reported in Italy [14–17].

A MenW:cc11 strain responsible for an outbreak associated with the Hajj pilgrimage at the turn of the century, became endemic in Europe where it persisted for several years [18]. The outbreak strain also disseminated into Africa, and then later to several South American countries [19,20]. In 2009, this strain began causing disease in the UK (the original 2009 UK strain). This clone is indistinguishable from the original Anglo-French Hajj associated strains by conventional MLST and antigen fine typing methods, and is recognised as MenW:cc11:P1.5,2:F1-1 [5,21]. A novel variant of the original 2009 strain (novel UK 2013 strain) was responsible for an outbreak among scouting enthusiasts following their return from the Japanese 2015 scout jamboree [22,23]. This clone is now endemic in some European countries including the UK, France, Sweden, and the Netherlands [24–27].

The highly clonal nature of the cc11 lineage makes the recognition of clonal variants challenging. MLST recognises virtually all invasive cc11 strains as sequence type 11 (https://pubmlst.org/neisseria/), the majority of which are homogeneous for the PorA outer membrane protein (OMP), and type as P1.5,2 using mono clonal antisera. Antigen fine-typing too is unsatisfactory, distinguishing among some MenC:cc11 clones, but lacking the necessary discriminatory power to establish the true phylogenetic relationships among contemporary circulating clones, or to historically significant strain types.

The clone associated with the international MenC epidemic of the 1990s and early 2000s is recognised by MLEE as electrophoretic type 15 (ET-15), and is characterised by a point mutation in the fumerase gene, or by the presence of insertion sequence element IS1301 [28,29].

In response to increasing MenC incidence, the Republic of Ireland (RoI) introduced the meningococcal C conjugate (MCC) vaccine into the routine infant schedule (October 2000), and offered the vaccine on a one-off basis to all those under 23 years. Subsequently, MenC incidence decreased from an incidence rate (IR) = 4.37/100,000 population (164 cases) in 1999/2000, to IR = 1.68/100,000 (64 cases) in 2000/2001 (OR 0.58, 0.42-.80, p<0.002), and had further decreased to just 20 cases by 2001/2002 [30].

Over the 2002/2003 to 2012/2013 period, the average annual MenC incidence was 0.08/100,000. This increased to 0.34/100,000 during the 2013/2014 to 2017/18 period. Incidence peaked in 2016/17 reaching 0.72/100,000 with an associated case fatality rate of 14.7%. MenW disease incidence was 0.02/100,000 in 2013/2014, and has increased each year since then to 0.29/100,000 in 2017/18, with an associated case fatality rate of 28.6% (www.hpsc.ie).

To understand if recent increases in MenC incidence are due to the emergence of a novel clone, or the re-emergence of clones prevalent at the time of the introduction of MenC vaccine, we characterised IMD-associated MenC:cc11 isolated during the 1997/98 to 2016/17 epidemiological years by WGS. We also compared these genomes to international invasive MenC: cc11 genomes. To understand the nature of the currently circulating MenW strains, all IMD-associated MenW isolates collected between 2010/11 and 2016/17 were compared to international examples of the original Hajj clone, and the more recently emerged original 2009 UK, and novel 2013 UK lineages. MenC and MenW strains isolated during a recent national carriage study among university students were also included.

## Materials and methods

In the Republic of Ireland (RoI) all invasive cases of meningococcal disease are reported to the Health Protection Surveillance Centre (HPSC) and the corresponding isolates and/or disease associated specimens are sent to the Irish Meningitis and Sepsis Reference Laboratory (IMSRL). *N*. *meningitidis* confirmation is by (PCR and real-time PCR) [31], and strain characterisation methods include serogroup [32], PorA and FetA variable region sequencing [33,34], multi-locus sequence typing (MLST)[5], and multi locus restriction typing (MLRT) [35].

### Study isolates

From a national collection of previously characterised IMD-associated isolates, 74 invasive MenC strains isolated during the 1997/98 to 2016/17 epidemiological years were included in this study.

All invasive meningococci isolated since 2010/11 have been characterised by WGS and were included in the study (n = 31). Prior to 2010/11, we retrospectively identified MenC:cc11 isolates, and characterised these isolates by whole genome sequencing. Clonal complex was inferred from MLRT characterisation, which recognises cc11 meningococci as restriction type 1 (RT-1). All invasive MenC isolates with RT-1 identified between 2001/02 to 2010/11 were included (n = 23). During the 1997/98 to 2000/2001 epidemiological years, a period of high incidence, 110 MenC-RT-1 isolates were identified, and 20 were included in the study. For these isolates PorA sequence type data was used as a proxy for genotype diversity, and isolates were chosen to broadly represent genotype diversity. Also included were asymptomatically carried MenC strains (n = 16) isolated from university students between November 2016 and March 2017. In all 90 MenC isolates were analysed. The national carriage study was ethically approved by the Children’s University Hospital ethics committee, and conducted in a fully anonymised manner where no participant identifiable information was collected.

Thirty-three MenW isolates were also included; 18 invasive strains isolated between 2010/11 to 2016/17, and 15 carriage strains isolated during the 2016/17 national carriage study.

### International MenC and MenW comparisons

The PubMLST *Neisseria* database was used to compare MenC:cc11 study isolates (n = 90) with invasive European MenC:cc11 strains (n = 689). In total 779 isolates (1997 to 2017) were compared; UK (n = 362), Italy (n = 147), France (n = 110) and the RoI (n = 90), Sweden (n = 27), Finland (n = 11), Spain (n = 10), Slovenia (n = 7), Malta (n = 5), Iceland (n = 5), Greece (n = 3), Croatia (n = 1) and Poland (n = 1).

MenW:cc11 isolates (n = 26) from the RoI were compared to an international collection of MenW:cc11 isolates, chosen to represent examples of the original Hajj strain (n = 170), and the more recent variants, the original UK 2009 and novel 2013 UK strains (n = 890). The isolate distribution by country was as follows: the UK (n = 878), the Netherlands (n = 121), France (n = 5), Sweden (n = 5) and Greece (n = 2).

### Genome sequencing

Genomic DNA was sequenced using the Illumina HiSeq platform (Oxford Genomics Centre, Wellcome Trust Centre for Human Genetics, University of Oxford, United Kingdom). Short read sequences were assembled *de novo* using the VelvetOptimiser algorithm [36], and then added to the Bacterial Isolate Genome Sequence Database (BIGSdb) on the Neisseria PubMLST website (https://pubmlst.org/neisseria/) [37]. The genomes were automatically scanned and annotated using alleles previously defined in the sequence definition database. The number of contigs assembled per genome ranged from 107 to 322, with a median of 142. The corresponding contig total lengths were between 2.11 and 2.24 Mb, with a median of 2.14 mega bases.

### Phylogenetic analysis

Genome comparisons were made using a gene-by-gene approach, where the ‘Genome Comparator’ tool identifies and assigns a numerical designation to each unique allele encountered [37]. The core genome MLST (cgMLST) scheme, which utilises a set of 1605 loci, was chosen as the basis of all genome comparisons [6]. The resultant distance matrix was visualised as a Neighbour-Net network in SplitsTree (version 4.14.4) [38,39]. Phylogenetic clusters were labelled consistently with a large international study of cc11 meningococci, which identified two main branches termed 11.1 and 11.2 [40]. Statistical tests were carried out in *R* (version 3.5.1, www.r-project.org).

### Antimicrobial resistance

For isolated collected since the 2012/13 epidemiological, minimum inhibitory concentrations were determined for penicillin, rifampicin, and ciprofloxacin by the E-Test method, and using Muller-Hinton agar supplemented with 5% sheep’s blood [41]. Phenotypes were assigned in accordance with EUCAST guidelines [41].

## Results

### Incidence of MenC and MenW disease

**Fig 1:**
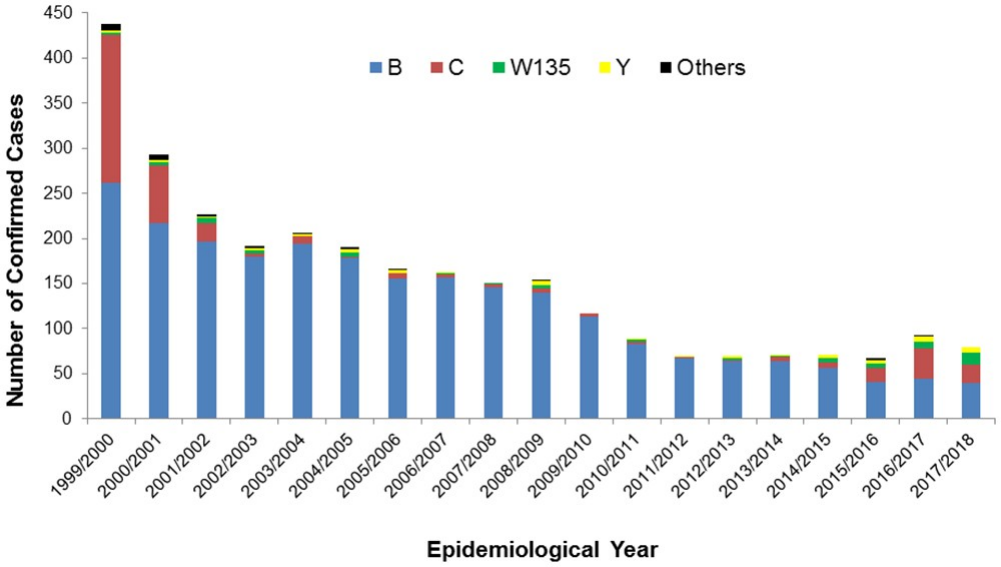
Confirmed incidence of IMD by serogroup in the Republic of Ireland, during the 1999/2000 to 2017/18 epidemiological years. A reduction in MenC IMD is observed in the years immediately preceding the meningococcal conjugate C vaccine introduction (October 2000). MenC disease incidence remains low for several years before increasing again during the most recent epidemiological years, and concomitant with increases in MenW disease incidence.

The predominant serogroups associated with meningococcal disease in the RoI are B, W, C, and Y (Fig 1). Serogroup B disease incidence has declined since the turn of the century. Disease incidence associated with serogroups W and C have increased since 2013. The number of associated deaths by serogroup has also increased (Fig 1).

**Fig 2:**
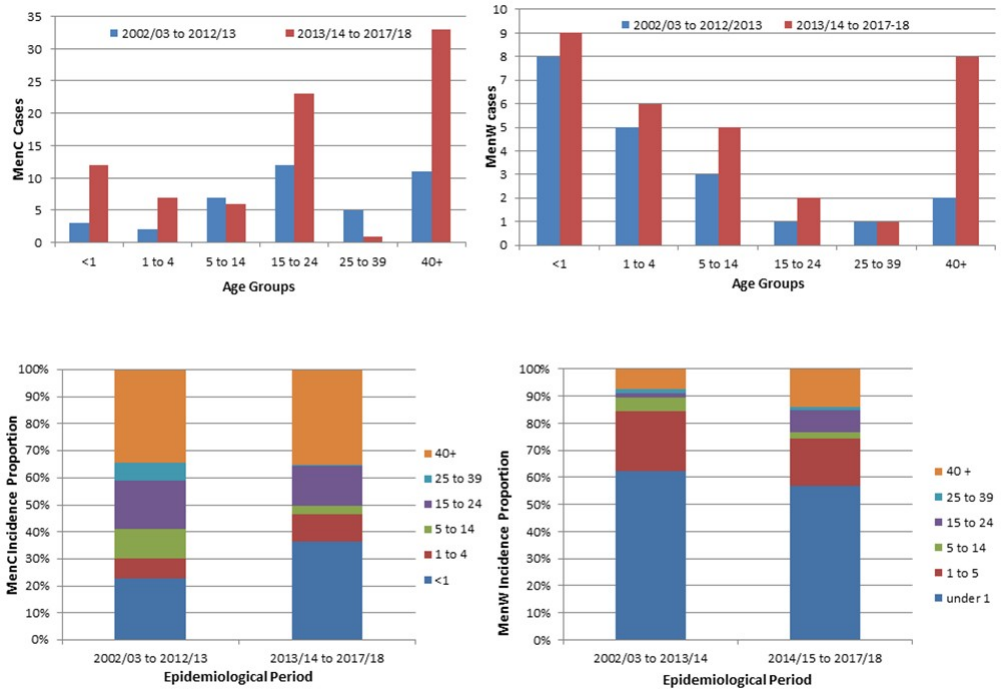
Actual numbers and proportions of both MenC and MenW disease incidence, by age group and over the epidemiological periods 2002/03 to 2012/13 and 2013/14 to 2017/18. The number of cases of MenC and MenW disease are shown in the top row, and age specific proportions of MenW and MenC incidence is shown in the bottom row.

The average MenC incidence rate increased significantly between the periods 2002/03–2012/13 (0.08 cases per 100,000 population) and 2013/14–2017/18 (0.34 cases per 100,000 population). The median age of those with MenC disease varied significantly between these periods (2-sample t-test, p<0.01); between 2002/03 to 2012/13 the median age for MenC incidence was 10.8 years (Quartile 1 (Q1)—2.7, Quartile 3 (Q3)—18.9), and during 2013/14 to 2017/18 the median MenC incidence age increased to 18.8 years (Q1–6.7, Q3–57.8). The case fatality rate (CFR) increased from 7.7% (n = 3/39) for the period 2002/03–2012/13, to 11.2% (n = 9/80) during 2013/14–2017/18 (p = 0.75). Age specific MenC incidence increased non-uniformly between these periods, with the largest proportional increases observed in in infants (from 23% to 36%, non-significant, 2-tailed fisher’s exact test) (Fig 2).

The average MenW incidence increased between the period 2002/03–2012/13 (0.04 cases per 100,000 population), and 2013/14–2017/18 (0.16 cases per 100,000 population). Non-uniform changes in age specific incidence were observed between these periods; an increase from 0.34/100,000 to 2.05/100,000 was observed in 15–24 year olds, and from 1.59/100,000 to 3.61/100,000 in those over 40 years (Fig 2). These changes were not statistically significant. The median age of MenW cases increased from 1.71 years (Q1–0.64, Q3–7.64) during the 2002/03 to 2012/2013 period, to 9.8 years (Q1–0.96, Q3–43.0) during the 2013/14 to 2017/18 period (2 sample t-test, p = 0.06). Over the same periods, the average yearly MenW CFR increased from 5% (n = 1/20) to 13.9% (n = 5/36). The highest yearly rate observed was 28.6% (n = 4/14, July 2017 to June 2018). Before 2012/13, MenW disease isolates were associated with cc22 exclusively (data not shown), and since then cc11 MenW isolates have predominated (S1 Fig).

### Population structure of MenC and MenW isolates

**Fig 3:**
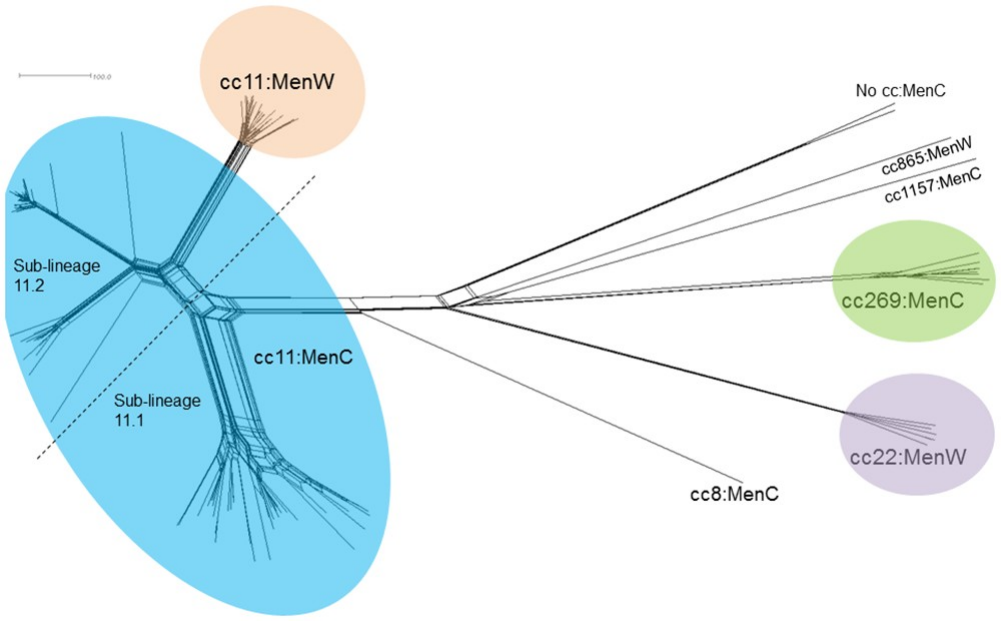
cgMLST NeighbourNet diagram showing the population structure of MenC and MenW disease isolates from the RoI (n = 135). The majority of isolates, 86% (n = 116/135) were cc11; 90 MenC (74 invasive and 16 from carriage) and 26 MenW isolates (14 invasive and 12 from carriage). The remaining 19 isolates were identified as members of cc269 (n = 8), cc22 isolates (n = 6), cc865 (n = 1) and cc1157 (n = 1) complexes, and two isolates were unassigned to a clonal complex. The scale bar represents a pairwise allelic difference of 100.

The study included 134 isolates; 101 MenC isolates (n = 75 invasive, n = 26 carriage), and 33 MenW isolates (n = 18 invasive, and n = 15 carriage). The population structure of these isolates was determined by core genome MLST (cgMLST) which compares isolates gene-by-gene (Fig 3). The majority of invasive MenC isolates (n = 74/75) and carried MenC isolates (n = 16/26) were cc11. The remaining invasive MenC isolate (ST-1434) could not be assigned to a clonal complex, and was paired with a carriage isolate of the same sequence type, and appeared distinct and isolated from other lineages. The remaining MenC carriage isolates were either cc269 (n = 8) or cc1157 (n = 1).

The majority of invasive (n = 14/18) and carried (n = 12/15) MenW isolates were cc11. Also observed were invasive (n = 3) and carried (n = 3) cc22:MenW isolates, a single invasive cc865:MenW, and a single carried cc269:MenW isolate.

Non-cc11:MenC and non-MenW:ccc11 isolates will not be discussed further.

### Phylogenetic comparison MenC:cc11 isolates

**Fig 4:**
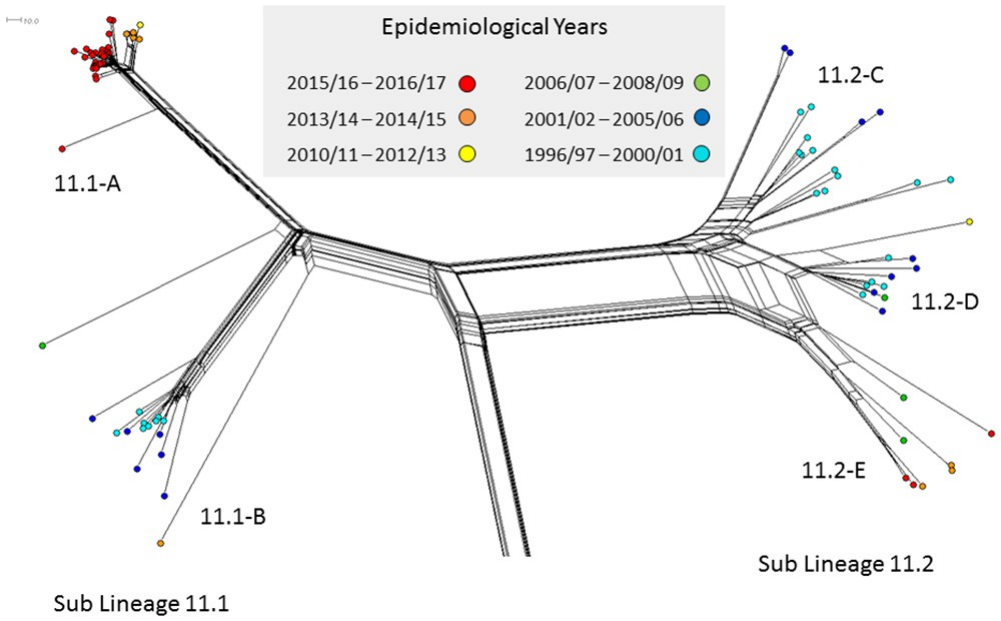
cgMLST NeighbourNet diagram of cc11MenC isolates from disease cases (n = 74, 1997–2017) and nasopharyngeal carriage (n = 28, 2016/17) in the RoI. Colours indicate different periods of isolation. Scale bar represents a pairwise allelic difference of 10.

Genomic comparisons of 90 cc11 MenC genomes (74 invasive and 16 carried isolates) formed a bifurcating structure composed of two main branches 11.1 and 11.2 (Fig 4). The sub-lineage 11.1 has two distinct groupings (11.1-A and 11.1-B), in which isolates tended to cluster more tightly, as compared to sub-lineage 11.2, which contained three less distinct groupings (11.2-C, 11.2-D, and 11.2-E). All sub-lineage 11.2 isolates carried the *fumC* 640 G—>A polymorphism characteristic of the ET-15 meningococci, and were generally composed of strains isolated pre-MenC vaccine introduction. Greater antigenic diversity was observed among sub-lineage 11.2 isolates (S1 Table). The majority of all cc11 isolates (n = 88/90) shared the same sequence type, ST-11. A single example of ST-13179 and ST-13990 was observed.

#### Sub-lineage 11.1 group-A

All 38 isolates (22 invasive, 16 carriage) of sub-lineage 11.1 group-A shared the same strain type (ST-11:P1.5,2:F3-3), and formed two distinct temporally structured clusters (Fig 4). The orange cluster contained half of invasive meningococcal strains isolated between the epidemiological years 2013/14 and 2014/15 (85%, n = 5/10). These isolates varied at an average of 32 of the 1605 cgMLST loci. The second red cluster contained invasive MenC:cc11 meningococci isolated between 2015/16 and 2016/17, and accounted for the majority of meningococci isolated during this period (78%, n = 14/18). On average these isolate genomes varied at 17 cgMLST loci. All 16 asymptomatically carried MenC:cc11 isolated during the 2016/17 epidemiological year clustered in this branch, and were interspersed among the invasive isolates. The sub-lineage 11.1 group-A phylogeny was consistent with a recent clonal expansion.

#### Sub-lineage 11.1 group-B

All 14 isolates comprising sub-lineage 11.1 group-B were characterised as ST:11:P1.5,2. Temporal structuring was again evident; the majority of strains clustering in the central region were isolated between the 1996/97 and 2000/01 epidemiological years (teal), whereas most diversified strains were isolated between the 2001/02 to 2004/05 epidemiological years (blue).

#### Sub-lineage 11.2

Sub-lineage 11.2 group-C strains (n = 16) were isolated between the 1996/97 and 2005/06 epidemiological years (teal), and was the only group to exhibit variation at the porA variable regions; P1.5,2 (n = 9), P1.5–2,10 (n = 2) and P1.5–1,10–4 (n = 2) and P1.18–2, 26–2 (n = 1). The FetA peptide F3-6 was predominant (n = 12/16) (S1 Table). Eight of the 12 sub-lineage 11.2 group-D strains were characterised as ST:11:P1.5–1,10–8:F3-6, and were isolated between the 1999/00 and 2010/11 epidemiological years. Sub-lineage 11.2 group-E isolates (n = 8) shared the same type (ST-11:P1.5–1,10–8:F3-6) and were isolated between the 2006/07 and 2016/17 epidemiological years, six of which were isolated in the most 3 recent epidemiological years. These latter strains are likely descendants of strains circulating at and around the time of the MenC vaccine introduction. This particular sub-lineage 11.2 group-E has been associated with invasive disease among MSM in Europe and the US [42,43], and in urethritis in homosexual men in the US [42,44]. Most of the sub-lineage 11.2 group-E isolates studied here harbour intact and in frame *aniA*, which may allow the organism to adapt to the anaerobic environment of the urogenitary tract (S1 Table) [44].

#### RoI MenC:cc11 isolates in the context of international MenC:cc11 strains

Irish MenC isolates were compared to a set of 779 contemporarily circulating strains in Europe (S2 Fig). The greater sample size of the international collection reveals the cc11 population structure in greater detail. Despite the disparity in sample size between the two isolate collections, the same basic MenC:cc11 population structure is observed, which indicates that the Irish isolates chosen from this period are representative of diversity circulating in Europe during the studied period and the robustness of the sub-lineage differentiation. The temporal distribution of the European isolates is consistent with the RoI isolate pattern, which suggests that MenC:cc11 strains have spread frequently between these countries. The comparison also shows that the dominant MenC clone circulating at present in the RoI is not unique to the RoI.

#### Phylogenetic comparison MenW:cc11 isolates

**Fig 5:**
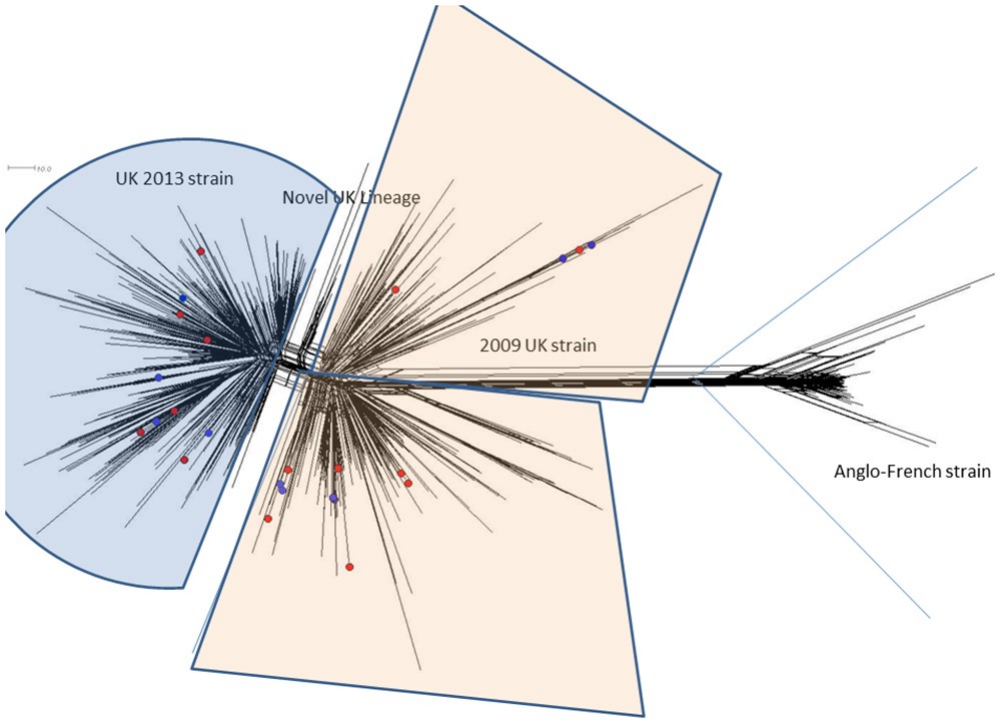
cgMLST NeighbourNet diagram of Irish MenW:cc11 isolates and representative examples of European MenW:cc11 isolates (unlabelled). Unlabelled isolates were chosen and represent the cc11 lineage structure established in 2015 (Lucidarme *et al*), and included examples of the original Hajj strain (Anglo-French strain), and the more recent UK 2009, and novel UK 2013 strains. Irish disease associated cases (n = 16, 2010–2017) are highlighted in red, and nasopharyngeal carriage isolates (n = 12, 2016/17) are highlighted in blue, and are observed interspersed among both the UK 2009 and novel 2013 UK strain clusters. There were no examples among the Irish study isolates of the original Hajj strain. Scale bar represents a pairwise allelic difference of 10.

Twenty-six MenW:cc11 meningococci were isolated between the 2010/2011 and 2016/17 epidemiological years. To determine the identity of these isolates, the invasive (n = 14) and carriage isolates (n = 12) were compared to examples of the original Hajj strain, the original 2009 UK strain, and the most recent variant, the novel UK-2013 strain by cgMLST (Fig 5). The Irish invasive and carriage isolates were generally interspersed among the comparison isolates. Seven invasive and seven carriage isolates were observed among the original 2009 UK cluster, while the remaining 7 invasive and 5 carriage isolates were observed among the novel UK 2013 variant. No examples of the original Hajj strain were observed.

#### Vaccine and antimicrobial resistance antigens

Among cc11 MenC and cc11 MenW isolated since 2014/15 epidemiological year (including carriage cc11 isolates) two Bexsero Antigen Sequence Types (BAST) dominated; BAST 8 (PorA 5,2; fHbp 22; NHBA 29; NadA 29) among MenC isolates, and BAST 2 (PorA 5,2; fHbp 22, NHBA 29, NadA 6) among MenW isolates. Antimicrobial resistance genes for ciprofloxacin, penicillin, and rifampicin (*gyrA*, *penA*, and *rpoB* respectively) were homogeneous among these isolates, and were consistent with isolates sensitive phenotypes (S1 Table).

## Discussion

In the RoI, disease incidence and associated case fatality rates due to MenC and MenW meningococci have increased in recent years. We have used WGS to investigate the isolates associated with these increases to resolve the uncertainty arising from conventional molecular typing method’s limited capacity to discriminate among cc11 sub-lineages. This study comprehensively characterised invasive MenC and MenW isolates by WGS, and compared genomes using cgMLST (n = 1605 loci).

Comparisons showed the invasive and carried MenC and MenW isolates studied were predominantly cc11. The majority of MenC incidence over the last five epidemiological years was due to a clone genetically distinct from the ET-15 MenC:cc11 epidemic strains. These isolates comprised two temporally structured clusters of highly similar clones. The emergence and expansion of this clone coincides with increasing MenC disease incidence observed since 2013. The interspersion of the carriage and disease isolates indicate that disease associated with this clone is largely a consequence of transmission. These isolates lacked the ET-15 *fumC* mutation, and were highly homologous at important antigenic loci such porA and other 4CMenB antigen targets, but would not be anticipated as susceptible to Bexsero elicited antibodies.

A minority (21%, 6/28) of invasive isolates from recent epidemiological years were found in sub-lineage 11.2 (red and orange). Given their variation, these strains appear more similar at a genomic level to the pre-MenC vaccine strains. So far, sub-lineage 11.2 group-E genotypes have been limited to causing sporadic disease only. While these strains must still circulate, no examples were isolated in the 2016/17 carriage study, indicating that they are circulating at a lower frequency than sub lineage 11.1 group-A strains; or less likely, they colonise the nasopharynx to a density undetectable by the sampling technique employed in the carriage study.

International comparisons of Irish and European MenC:cc11 isolates showed that while recent disease in the RoI is caused by a distinct MenC:cc11 clone, it is not unique to the RoI. This comparison further confirms the capacity of hyper invasive MenC:cc11 strains to disseminate and spread internationally, an inherent characteristic of MenC:cc11 strains.

Taken together, the significant increase in MenC disease incidence from a novel disease genotype, the higher associated case fatality rates, the increase in median incidence age, and the potential for capsule switching events to occur in this lineage, are all reasons to warrant concern, and highlight a changing epidemiological pattern that will require continuous and careful monitoring, if not immediate action [10, 40].

The success of meningococcal conjugate C (MCC) vaccination programs is attributed to the vaccines capacity to elicit mucosal epithelial secretions containing anti-MenC antibodies, which can impact the carriage state and slow transmission [45]. Meningococcal carriage is age dependant, where the highest rates of are in teenagers and young adults [46]. Immunisation of this cohort disrupts MenC transmission and protects other non-vaccinated cohorts indirectly (herd immunity) [45,46]. Therefore, maintaining antibody titres among this cohort are essential to sustain the herd effect.

The longevity of MCC vaccination protection is unknown, although UK data had predicted a reduction of MenC disease for >15 years following vaccination [47]. A recent UK study reported that just 19% of 10 year olds immunised at 14 months with a single dose of MenC had protective antibody titres 9 years later [48]. While no similar seroprevalence data is available in the RoI, it is probable that MenC increases are due to waning herd immunity in the Irish population. Further, MCC herd immunity has likely reduced naturally acquired immunity to MenC strains as a consequence of reducing MenC transmission [48,49]. When MCC herd effects fail, transmission is likely to increase, and an immunologically naïve population (compared to a pre vaccine levels), might be at higher risk to MenC disease.

Prior to 2011 in the RoI, no examples of the characteristic Hajj genotype (MenW:cc11:1.5,1.2) have been identified among either disease isolates or associated diagnostic extracts. Therefore, disease incidence associated with the original Hajj clone was either rare or absent before 2011. The data presented here shows that since then, cc11 meningococci were responsible for the majority of invasive MenW disease. These isolates, and asymptomatically carried strains, were identified as the original 2009 UK and novel 2013 UK strain clones, and show that these strain types are now endemic in the RoI. The original Hajj clone however, was not observed in either carriage or disease. Why the original Hajj strain did not become endemic and the current strains have is unclear.

The year by year incidence increases, and the high CFR rates associated with this hyper virulent MenW strain reported here are consistent with reports from other countries [24–26,50,51,52]. In addition, reports of atypical clinical presentations have been associated with this strain, such as vomiting, diarrhoea, and necrotising fasciitis and septic arthritis and pericarditis [24,26,53]. We have clearly established that the majority of Irish MenW isolates are indistinguishable from those strains driving MenW disease incidence in the UK, Netherlands, Sweden, Italy and France. Further, we also report isolating this clone from asymptomatic carriage in University students, confirming that this clone is now endemic in the RoI.

## Summary and conclusions

Unambiguous characterisation of bacterial isolates is essential to establishing relationships among bacterial isolates nationally and internationally, and relating contemporarily circulating strains to historically significant strain types. This analysis is invaluable to informing public health decisions to adapt vaccination policy to counter changes in epidemiological patterns. We have shown that the highly clonal nature of cc11 meningococcal strains can be comprehensively resolved by WGS characterisation and publicly accessible tools. This study also highlights the importance of regular carriage studies; relating disease associated genotypes to circulating carriage genotypes can help establish the degree to which novel or emerging disease associated genotypes have become endemic.

The national immunisation advisory committee (NIAC) are currently (April 2019) considering a change in vaccine policy in response to changing MenC and MenW epidemiological patterns the RoI. Data from this study clarifying the nature of recent meningococcal epidemiological changes was used to inform this pending decision.

## Supporting information

S1 Fig Disease associated MenW isolates by clonal complex. Disease associated MenW strains isolated in the republic of Ireland between 2013 and 2017, characterised by MLST clonal complex.
(TIF)

S2 Fig cgMLST NeighbourNet diagram showing the population structure of European MenC disease strains (n = 779) isolated over the study period. This is a SplitsTree NeighbourNet diagram of 779 invasive cc11:MenC strains isolated in Europe between 1997 and 2017; the UK (n = 362), Italy (n = 147), France (n = 110), Ireland (n = 90), Sweden (n = 27), Finland (n = 11), Spain (n = 10), Slovenia (n = 7), Malta (n = 5), Iceland (n = 5), Greece (n = 3), Croatia (n = 1) and Poland (n = 1). While not wholly representative of European strains over the study period, this larger cc11:MenC isolate comparison reveals the cc11:MenC population structure in greater detail. The overall phylogeny of the European isolates is largely congruent with that of Fig 4 (which shows Irish isolates only). Sub-lineage period of isolation is also consistent with those of Fig 4. This shows that the MenC strains currently circulating in the RoI are not unique to the RoI.
(TIF)

S1 Table Molecular characteristics of clonal complex 11 study isolates. Table showing clonal complex 11 isolates and various molecular characteristics including meningococcal fine-types, BAST types, and antimicrobial genotype.
(XLSX)

S2 Table Table showing all study isolate BIGS ID and associated ENA accession number. The isolate genome assemblies used in the study can be found in the Bacterial Isolate Genome Sequencing database (BIGS_DB_) using the BISGS ID numbers. The short reads associated with each genome assembly have been deposited at the European Nucleotide Archive (ENA) filed under the corresponding ENA accession number.
(DOCX)
