# Supplementary figures and images for: cgMLST characterisation of invasive Neisseria meningitidis serogroup C and W strains associated with increasing disease incidence in the Republic of Ireland
Source: PLoS One. 2019 May 29;14(5):e0216771. doi: 10.1371/journal.pone.0216771 (PMC6541471; doi:10.1371/journal.pone.0216771)

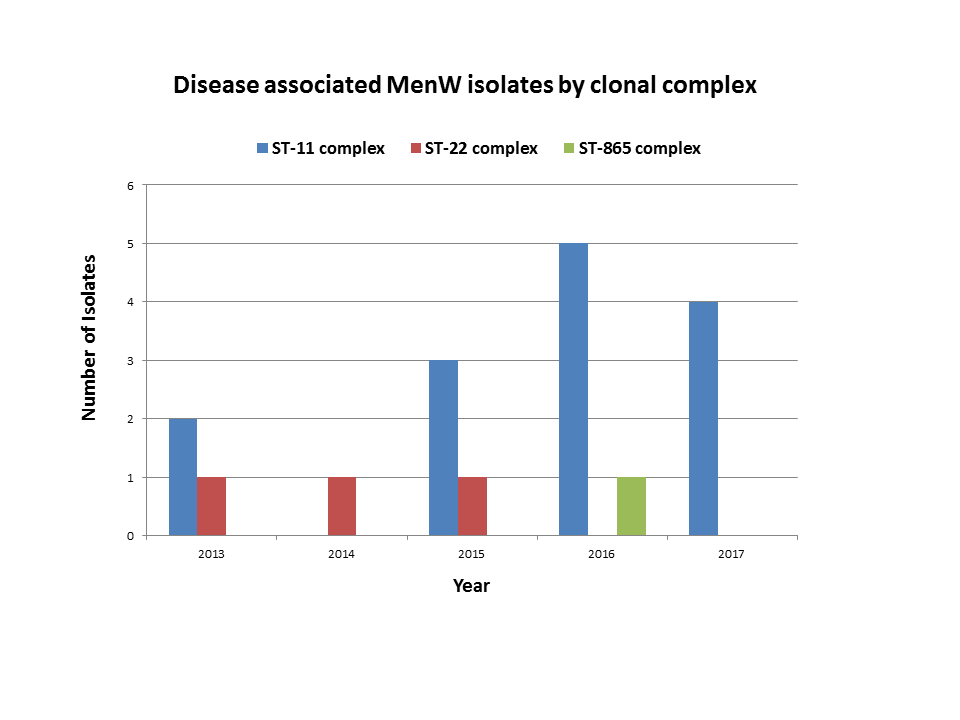

Supplement: S1 Fig — Disease associated MenW strains isolated in the republic of Ireland between 2013 and 2017, characterised by MLST clonal complex. (TIF) [file pone.0216771.s001.tif]

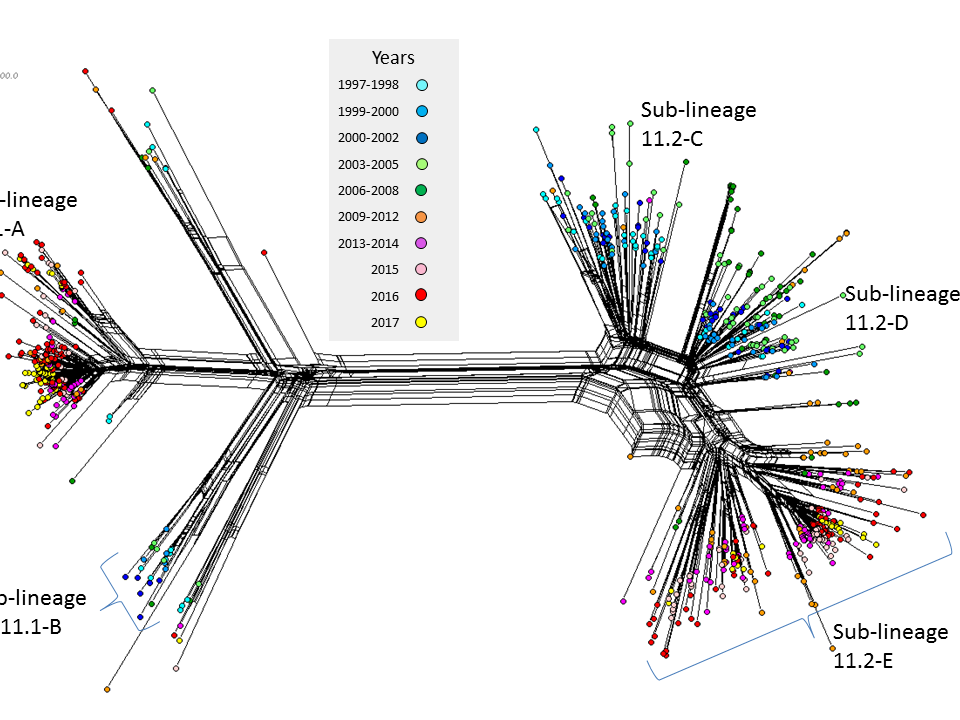

Supplement: S2 Fig — This is a SplitsTree NeighbourNet diagram of 779 invasive cc11:MenC strains isolated in Europe between 1997 and 2017; the UK (n = 362), Italy (n = 147), France (n = 110), Ireland (n = 90), Sweden (n = 27), Finland (n = 11), Spain (n = 10), Slovenia (n = 7), Malta (n = 5), Iceland (n = 5), Greece (n = 3), Croatia (n = 1) and Poland (n = 1). While not wholly representative of European strains over the study period, this larger cc11:MenC isolate comparison reveals the cc11:MenC population structure in greater detail. The overall phylogeny of the European isolates is largely congruent with that of Fig 4 (which shows Irish isolates only). Sub-lineage period of isolation is also consistent with those of Fig 4. This shows that the MenC strains currently circulating in the RoI are not unique to the RoI. (TIF) [file pone.0216771.s002.tif]
