# Supplementary material for: cgMLST characterisation of invasive Neisseria meningitidis serogroup C and W strains associated with increasing disease incidence in the Republic of Ireland
Source: PLoS One. 2019 May 29;14(5):e0216771. doi: 10.1371/journal.pone.0216771 (PMC6541471; doi:10.1371/journal.pone.0216771)
Supplement: S2 Table — The isolate genome assemblies used in the study can be found in the Bacterial Isolate Genome Sequencing database (BIGSDB) using the BISGS ID numbers. The short reads associated with each genome assembly have been deposited at the European Nucleotide Archive (ENA) filed under the corresponding ENA accession number. (DOCX) [file pone.0216771.s004.docx]

| **BIGS ID** | **ENA Accession** |
| --- | --- |
| 26824 | ERR351667 |
| 26825 | ERR351668 |
| 26859 | ERR351702 |
| 26898 | ERR351741 |
| 26900 | ERR351743 |
| 26914 | ERR351757 |
| 30694 | ERR654733 |
| 30698 | ERR654801 |
| 30727 | ERR654786 |
| 36138 | ERR976831 |
| 36156 | ERR976844 |
| 36817 | ERR957610 |
| 36821 | ERR957623 |
| 36822 | ERR957614 |
| 36823 | ERR957615 |
| 36824 | ERR957616 |
| 36825 | ERR957617 |
| 36827 | ERR957619 |
| 36828 | ERR957620 |
| 55208 | ERR2113305 |
| 55234 | ERR2113331 |
| 55304 | ERR2113401 |
| 55316 | ERR2113413 |
| 55331 | ERR2113428 |
| 55339 | ERR2113436 |
| 55346 | ERR2113443 |
| 55347 | ERR2113444 |
| 55354 | ERR2113451 |
| 55359 | ERR2113456 |
| 55364 | ERR2113461 |
| 55380 | ERR2113477 |
| 55383 | ERR2113480 |
| 55384 | ERR2113481 |
| 55393 | ERR2113490 |
| 55395 | ERR2113492 |
| 55447 | ERR2113544 |
| 55464 | ERR2113561 |
| 55465 | ERR2113562 |
| 55467 | ERR2113564 |
| 55478 | ERR2113575 |
| 55482 | ERR2113579 |
| 55490 | ERR2113587 |
| 55492 | ERR2113589 |
| 55495 | ERR2113592 |
| 55507 | ERR2113604 |
| 55509 | ERR2113606 |
| 55514 | ERR2113611 |
| 55522 | ERR2113619 |
| 55527 | ERR2113624 |
| 55530 | ERR2113627 |
| 55537 | ERR2113634 |
| 55538 | ERR2113635 |
| 55539 | ERR2113636 |
| 55541 | ERR2113638 |
| 55542 | ERR2113639 |
| 55543 | ERR2113640 |
| 55550 | ERR2113647 |
| 55551 | ERR2113648 |
| 55554 | ERR2113651 |
| 55562 | ERR2113659 |
| 55566 | ERR2113663 |
| 55567 | ERR2113664 |
| 55568 | ERR2113665 |
| 55569 | ERR2113666 |
| 55570 | ERR2113667 |
| 55572 | ERR2113669 |
| 55575 | ERR2113672 |
| 55576 | ERR2113673 |
| 55577 | ERR2113674 |
| 55578 | ERR2113675 |
| 55579 | ERR2113676 |
| 55581 | ERR2113678 |
| 55582 | ERR2113679 |
| 55583 | ERR2113680 |
| 55585 | ERR2113682 |
| 57382 | ERR2259045 |
| 57392 | ERR2259006 |
| 57399 | ERR2258991 |
| 57411 | ERR2259012 |
| 57429 | ERR2259048 |
| 57464 | ERR2258916 |
| 57471 | ERR2258925 |
| 57477 | ERR2258937 |
| 57485 | ERR2258952 |
| 57486 | ERR2258953 |
| 57497 | ERR2258892 |
| 57501 | ERR2259057 |
| 57503 | ERR2259068 |
| 57504 | ERR2259066 |
| 57518 | ERR2259043 |
| 57524 | ERR2258976 |
| 57525 | ERR2258974 |
| 57526 | ERR2258972 |
| 57527 | ERR2258970 |
| 57528 | ERR2258967 |
| 57529 | ERR2258965 |
| 57530 | ERR2258897 |
| 57531 | ERR2258931 |
| 57532 | ERR2258913 |
| 57533 | ERR2258912 |
| 57534 | ERR2258896 |
| 57535 | ERR2258945 |
| 57536 | ERR2259050 |
| 57537 | ERR2258915 |
| 57538 | ERR2259047 |
| 57541 | ERR2258906 |
| 57543 | ERR2258942 |
| 57544 | ERR2258899 |
| 57545 | ERR2258908 |
| 57546 | ERR2258929 |
| 57547 | ERR2258901 |
| 57548 | ERR2258940 |
| 57549 | ERR2258903 |
| 57550 | ERR2259061 |
| 57551 | ERR2259063 |
| 57552 | ERR2258938 |
| 57553 | ERR2258910 |
| 57554 | ERR2258894 |
| 57555 | ERR2258977 |
| 57556 | ERR2258979 |
| 57557 | ERR2258919 |
| 57558 | ERR2258935 |
| 57559 | ERR2258981 |
| 57560 | ERR2258933 |
| 57561 | ERR2258961 |
| 57562 | ERR2258963 |
| 57563 | ERR2258926 |
| 57564 | ERR2258960 |
| 57565 | ERR2258958 |
| 57566 | ERR2258924 |
| 57567 | ERR2258956 |
| 57568 | ERR2258922 |
| 57569 | ERR2258954 |
| 57570 | ERR2258928 |
| 57571 | ERR2258944 |
